# The IL-1 Antagonist Anakinra Attenuates Glioblastoma Aggressiveness by Dampening Tumor-Associated Inflammation

**DOI:** 10.3390/cancers12020433

**Published:** 2020-02-13

**Authors:** Max Hübner, David Effinger, Tingting Wu, Gabriele Strauß, Kristin Pogoda, Friedrich-Wilhelm Kreth, Simone Kreth

**Affiliations:** 1 Department of Anesthesiology, University Hospital, LMU Munich, 81377 Munich, Germany; max.huebner@med.uni-muenchen.de (M.H.); david.effinger@med.uni-muenchen.de (D.E.); tingting.wu@med.uni-muenchen.de (T.W.); gabriele.strauss@med.uni-muenchen.de (G.S.); 2 Walter-Brendel Center of Experimental Medicine, Faculty of Medicine, LMU Munich, 81377 Munich, Germany; kristin.pogoda@lrz.uni-muenchen.de; 3 Biomedical Center, Ludwig-Maximilians-University, 82152 Planegg, Germany; 4 Department of Neurosurgery, University Hospital, LMU Munich, 81377 Munich, Germany; friedrich-wilhelm.kreth@med.uni-muenchen.de

**Keywords:** glioblastoma, inflammation, IL-1β, anakinra

## Abstract

Background: The recombinant IL-1 receptor antagonist anakinra—currently approved for the treatment of autoinflammatory diseases—blocks IL-1β-mediated inflammatory signaling. As inflammation is a major driver of cancer, we hypothesized that anakinra might be able to mitigate glioblastoma (GBM) aggressiveness. Methods: Primary GBM or T98G cells were incubated alone or with peripheral blood mononuclear cells (PBMCs) and were subsequently treated with IL-1β and/or anakinra. T cells were obtained by magnetic bead isolation. Protein and mRNA expression were quantified by SDS-PAGE, qRT-PCR, and ELISA, respectively. Cell proliferation and apoptosis were analyzed via flow cytometry. Chemotaxis was studied via time-lapse microscopy. Results: Upon IL-1β stimulation, anakinra attenuated proinflammatory gene expression in both GBM cells and PBMCs, and mitigated tumor migration and proliferation. In a more lifelike model replacing IL-1β stimulation by GBM–PBMC co-culture, sole presence of PBMCs proved sufficient to induce a proinflammatory phenotype in GBM cells with enhanced proliferation and migration rates and attenuated apoptosis. Anakinra antagonized these pro-tumorigenic effects and, moreover, reduced inflammatory signaling in T cells without compromising anti-tumor effector molecules. Conclusion: By dampening the inflammatory crosstalk between GBM and immune cells, anakinra mitigated GBM aggressiveness. Hence, counteracting IL-1β-mediated inflammation might be a promising strategy to pursue.

## 1. Introduction

Inflammation has emerged as a major promoter of all stages of tumorigenesis [1,2,3]. Intricate networks of tumor-associated inflammation are driven by a perpetual crosstalk between cancer and tumor-infiltrating immune cells, which has mutually reinforcing effects and boosts the production of cytokine mediators by both immune and tumor cells [4,5,6]. The resulting inflammatory milieu not only promotes tumor progression, but also blocks anti-tumor immunity by impairing functions of the adaptive immune system [7,8]. Interleukin (IL)-1β has emerged as the master cytokine activating inflammatory signaling pathways in both cancer and immune cells [9,10,11]. In particular, in the tumor microenvironment (TME) of glioblastomas (GBM), IL-1β is extensively abundant [11,12,13]. Thus, blocking IL-1β might ameliorate the vicious cycle of self-aggravating inflammation and might reduce GBM aggressiveness [14,15]. In this scenario, the recombinant IL-1 receptor antagonist anakinra, routinely used in the treatment of autoimmune diseases, might hold potential as a promising new therapeutic approach [15,16]. 

In the current study, we addressed this issue in an in vitro model of the tumor microenvironment consisting of primary human GBM cells and human immune cells. We were able to provide evidence that anakinra exerts its effects in both GBM and immune cells: It puts the brake on tumor-promoting inflammatory signaling pathways in GBM and simultaneously promotes a shift towards an anti-inflammatory T cell phenotype without impairing anti-tumor immunity. As a result, anakinra diminishes GBM proliferation, migration, and invasion. Our findings indicate that administration of anakinra might be an interesting and novel therapeutic approach to reduce glioblastoma aggressiveness.

## 2. Results

### 2.1. Anakinra Dampened IL-1β-Induced Inflammatory Gene Expression in GBM and PBMC

**Figure 1:**
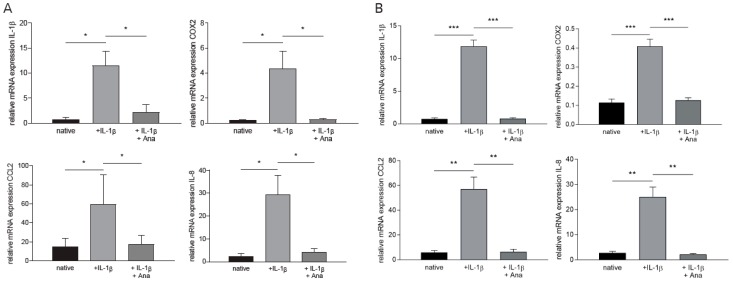
Anakinra dampened IL-1β-induced inflammatory gene expression in primary glioblastoma (GBM) cells and peripheral blood mononuclear cells (PBMCs). GBM cells and PBMCs were separately stimulated with IL-1β. Anakinra (Ana) was administered after 3h of incubation. mRNA expression levels of proinflammatory *IL-1β*, *COX-2*, *CCL2*, and *IL-8* were quantified by qRT-PCR. (A) mRNA expression of proinflammatory cytokines in GBM (n = 6, p < 0.041). (B) mRNA expression of proinflammatory cytokines in PBMCs (n = 5, *p* < 0.008). * *p* < 0.05, ** *p* < 0.01, *** *p* < 0.001.

We first assessed the impact of the “master cytokine” IL-1β on proinflammatory gene expression in primary GBM cells and peripheral blood mononuclear cells (PBMC) [17]. As shown in Figure 1, IL-1β stimulation resulted in a marked increase of the proinflammatory genes *IL-1β*, *COX-2*, *CCL2*, and *IL-8* in both GBM cells (Figure 1A: *IL-1β*: +13.73-fold ± 3.62, *COX-2*: +17.2-fold ± 5.65, *CCL2*: +2.90-fold ± 1.65, *IL-8*: +11.1-fold ± 3.64, n = 6, *p* < 0.041) and PBMCs (Figure 1B: *IL-1β*: +15.1-fold ± 1.31, *COX-2*: +2.57-fold ± 0.27, *CCL2*: +8.77-fold ± 1.63, *IL-8*: +8.22-fold ± 1.22, n = 5, *p* < 0.008). After an IL-1β stimulation period of 3h, administration of anakinra (1 µg/mL, dose-finding is depicted in Figure S1A,B) strongly diminished expression of these cytokines in both cell populations (Figure 1A,B: GBM: *IL-1β*: −80.6% ± 25.2%, *COX-2*: −92.3% ± 30.9%, *CCL2*: −70.3% ± 40.2%, *IL-8*: −85.7 ± 29.9%, n = 6; *p* < 0.041; PBMC: *IL-1β*: −93.3 ± 8.1 %, *COX-2*: −69.0% ± 7.9%, *CCL2*: −88.8% ± 15.9%, *IL-8*: −86.0% ± 11.8%, n = 5, *p* < 0.008).

Primary GBM, T98G, and U87 cells responded to anakinra treatment after IL-1β stimulation in a comparable manner (T98G/U87: Figure S1B,C). As the features of T98G cells provide a more life-like model of human GBM in contrast to U87 cells regarding their MGMT methylation status [18], primary GBM and T98G cells were mainly used for subsequent experiments.

### 2.2. Anakinra Inhibited IL-1β-Induced Tumor Proliferation and Migration

**Figure 2:**
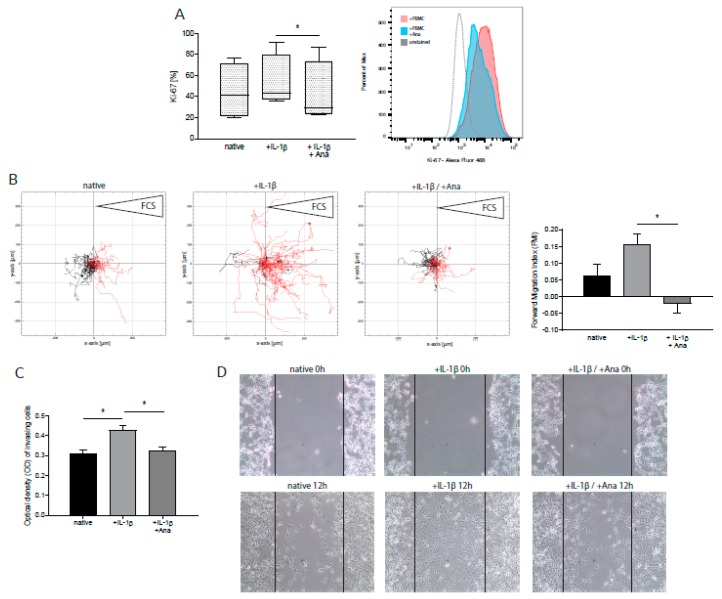
GBM proliferation, migration, and invasion, promoted by IL-1β stimulation, was mitigated after treatment with anakinra (Ana). T98G cells were stimulated with IL-1β in the presence or absence of anakinra. (**A**) Quantification of Ki-67-positive T98G cells by flow cytometry (n = 5, *p* = 0.027). One representative histogram is shown. (**B**) Analysis of chemotaxis time-lapse microscopy by single-cell tracking. IL-1β-stimulated T98G cells were incubated with FCS as a chemotactic stimulus (right reservoir). At least 40 cells were tracked. One representative example of four independent experiments is shown (left panel, n = 4). Analysis of forward migration index (FMI, coordinate of a cell in the indicated direction (x-axis) divided by the accumulated distance of its paths, representing efficiency of forward migration) (right panel, n = 4, *p* = 0.048). (**C**) Analysis of tumor cell invasion by transwell invasion assay. Stimulated T98G cells were seeded in cell culture inserts and allowed to migrate towards FCS as a chemotactic stimulus. Optical density of invasive T98G represents the amount of transmigrated cells (n = 3, *p* < 0.05). (**D**) 2D migration assay of GBM cells, stimulated with IL-1β in the presence or absence of anakinra at start and after 12h. Lines mark the initially cell-free area. A typical example of six experiments is shown (n = 6). * *p* < 0.05.

We next assessed the impact of IL-1β administration on GBM cell proliferation, migration, and apoptosis by intracellular flow cytometry and migration assay, respectively. Upon stimulation of GBM cells with IL-1β, we detected an increased proliferation rate as determined by Ki-67 protein levels [19] (Figure 2A: +19.8% ± 17.4%, n = 5, *p* = n.s.). This effect was significantly inhibited by administration of anakinra (Figure 2A: −21.0% ± 5.1%, n = 5, *p* = 0.027). Furthermore, we conducted time-lapse microscopy to assess the impact of IL-1β on GBM cell migration. As depicted in Figure 2B, an enhancement of directed migration towards a chemotactic stimulus was found (+1.49-fold % ± 0.98, n = 4, *p* = n.s.), whereas anakinra treatment blocked this pro-invasive effect of IL-1β (Figure 2B: −1.13-fold ± 0.37, n = 4, *p* = 0.048). Similar results were obtained using primary GBM cells (Figure S2). These findings were also corroborated in transwell invasion (Figure 2C) and 2D migration assays (Figure 2D), revealing a less invasive GBM phenotype after administration of anakinra. Tumor cell invasion was significantly enhanced by IL-1β (Figure 2C: +37.8% ± 2.9%, n = 3, *p* = 0.047), and anakinra markedly attenuated the invasive capacity of tumor cells (Figure 2C: −23.4% ± 2.7%, n = 3, *p* = 0.048).

IL-1β is known to act as an inhibitor of tumor apoptosis via the induction of signal transducer and activator of transcription 3 (STAT3) [11,20,21,22]. Concordantly, we found a trend towards IL-1β-induced induction of *STAT3* expression and reduced apoptosis rates in GBM cells. These effects were slightly attenuated by anakinra, however, without reaching statistical significance (Figure S3).

Collectively, these results suggest that two main features of GBM cell aggressiveness, proliferation and migration, are amplified by IL-1β-signaling, which can be prevented by anakinra administration in vitro.

### 2.3. Anakinra Dampened Inflammatory Crosstalk between GBM and Immune Cells, Resulting in a Less Aggressive GBM Phenotype

**Figure 3:**
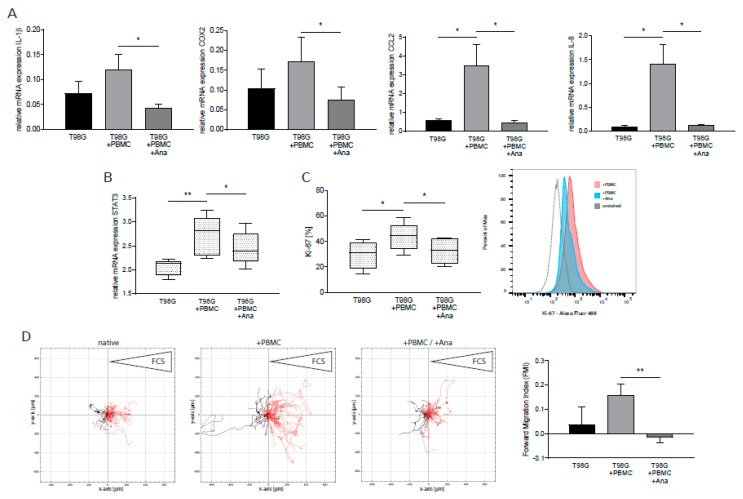
Anakinra inhibited co-culture-induced expression of proinflammatory cytokines and *signal transducer and activator of transcription 3 (STAT3)* in GBM and inhibits tumor proliferation and migration. T98G cells were indirectly incubated with PBMCs. (**A**) mRNA expression of proinflammatory cytokines in T98G (n = 7, *p* < 0.040). (**B**) mRNA expression of transcription factor *STAT3* in T98G (n = 7, *p* < 0.016). (**C**) Quantification of Ki-67-positive T98G cells by flow cytometry (n = 6, *p* < 0.05). One representative histogram is shown. (**D**) Analysis of chemotaxis time-lapse microscopy by single-cell tracking. T98G cells from indirect co-cultures were incubated with FCS as a chemotactic stimulus (right reservoir). At least 40 cells were tracked. One representative example of four independent experiments is shown (left panel, n = 4). Analysis of forward migration index (FMI, coordinate of a cell in the indicated direction (x-axis) divided by the accumulated distance of its paths, representing efficiency of forward migration) (right panel, n = 4, *p* = 0.008). * *p* < 0.05, ** *p* < 0.01.

We next aimed to investigate the effects of anakinra in a model mimicking the tumor microenvironment. To this end, we established a protocol of indirect co-cultivation of GBM cells and PBMCs under moderate hypoxic conditions (5% O_2_). Surprisingly, indirect co-incubation of GBM cells and PBMCs, without any additional stimuli, was sufficient to dramatically increase the expression of proinflammatory genes and *STAT3* in GBM cells (Figure 3A: *IL-1β*: +70.7% ± 32.4%, *COX-2*: +79.9% ± 51.8%, *CCL2*: +5.81-fold ± 2.04, *IL-8*: +14.11-fold ± 4.66, n = 7, *p* < 0.040; Figure 3B: *STAT3*: +26.9% ± 5.1%, n = 7, *p* = 0.006). Anakinra treatment was also able to abrogate induction of all genes investigated (Figure 3A: *IL-1β*: −64.0% ± 19.1%, *COX-2*: −56.3% ± 21.4%, *CCL2*: −87.1% ± 46.0%, *IL-8*: −91.7% ± 27.0%, n = 7, *p* < 0.040; Figure 3B: *STAT3*: −11.0 % ± 3.2%, n = 7, *p* = 0.015). We next assessed proliferation, migration, and apoptosis rates of T98G cells from co-culture experiments. As shown in Figure 3C and D, indirect contact with immune cells increased GBM proliferation rates (Figure 3C: +52.0% ± 17.5%, n = 6, *p* = 0.048) and enhanced directed tumor cell migration towards a chemotactic stimulus (Figure 3D: +3.36-fold ± 1.77, n = 4, *p* = n.s). Application of anakinra resulted in significantly reduced expression levels of Ki-67 (Figure 3C: −25.2% ± 6.8%, n = 6, *p* = 0.019) and, furthermore, was able to block the pro-migratory effects induced by GBM–PBMC co-culturing (Figure 3D: −1.09-fold ± 0.17, n = 4, *p* = 0.008). 

**Figure 4:**
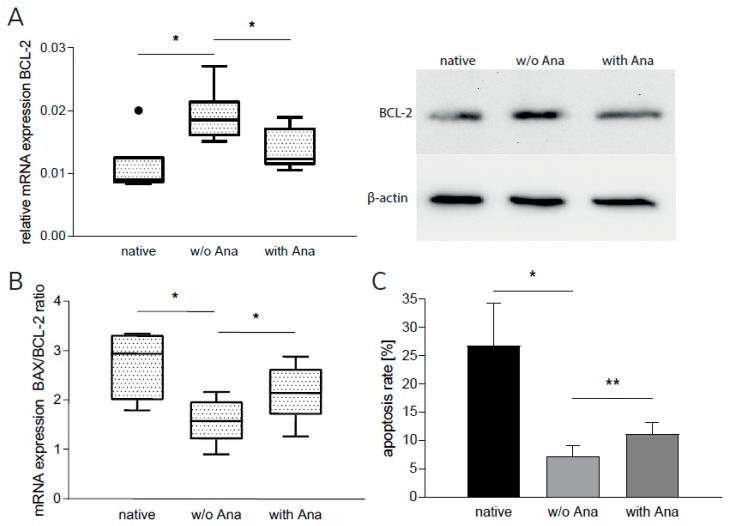
Anakinra increased apoptosis in GBM cells. T98G cells were incubated in conditioned media from direct GBM–PBMC co-cultures with or without (w/o) anakinra (Ana). (**A**) mRNA and protein expression of BCL-2 in T98G (n = 7, *p* < 0.035). One representative example of sodium dodecyl sulfate polyacrylamide gel electrophoresis is shown. (**B**) *BAX* and *BCL-2* mRNA expression in T98G were quantified in qRT-PCR and the BAX/BCL-2 ratio was calculated (n = 3, p < 0.05). (**C**) Percentage of apoptotic T98G cells as measured by flow cytometry (n = 7, *p* < 0.049). * *p* < 0.05, ** *p* < 0.01.

However, apoptosis of GBM cells was not affected by indirect co-culturing (data not shown), which led us to assume that direct cell–cell contact might be needed for induction of these signaling pathways. To this end, we directly co-cultured GBM cells with PBMCs. To deal with the difficulty of separating tumor cells from immune cells after direct incubation, we proceeded in two steps: (1) We harvested the supernatant of these direct co-cultures and (2) subsequently subjected it to GBM cell cultures. After an incubation time of 24 hours, we determined antiapoptotic BCL-2 mRNA and protein (densitometric analysis) expression, mRNA expression of proapoptotic BAX, and apoptosis rate. Incubation with supernatant from untreated co-cultures increased mRNA and protein expression of BCL-2 in native T98G cells (Figure 4A: mRNA: +76.9% ± 7.7%, n = 6, *p* = 0.031; protein: +20.3% ± 14.2%, n = 3) and reduced the *BAX/BCL-2* ratio, an established parameter determining the response to possible pro- or anti-apoptotic stimuli [23,24] (Figure 4B: −47.6% ± 9.1%, n = 3, *p* = 0.015). Concordantly, T98G apoptosis rate was reduced (Figure 4C: −63.7% ± 23.0%, n = 6, *p* = 0.048). When incubated with anakinra-treated direct co-culture supernatant, T98G cells displayed significantly diminished mRNA and protein levels of BCL-2 (Figure 4A: mRNA: −18.3% ± 7.5%, n = 6, *p* = 0.013; protein: −22.5% ± 3.5%, n = 3). The *BAX/BCL-2* ratio was induced significantly (Figure 4B: +36.5% ± 13.1%, n = 3, *p* = 0.049). In line with these findings, the percentage of apoptotic GBM cells, as measured by flow cytometry, was increased (Figure 4C: +43.6% ± 8.4%, n = 6, *p* = 0.002).

Taken together, these results provide evidence that the presence of PBMCs alone is sufficient to induce a proinflammatory phenotype in GBM cells, which in turn enhances GBM cell proliferation and migration and inhibits apoptosis. In contrast, treatment with anakinra attenuates inflammatory gene expression, inhibits proliferation, attenuates migration, and induces apoptosis. Thus, anakinra treatment induces a more benign GBM cell phenotype.

### 2.4. Anakinra Reduced Inflammatory Signaling in Lymphocytes Co-Cultured With GBM Cells

**Figure 5:**
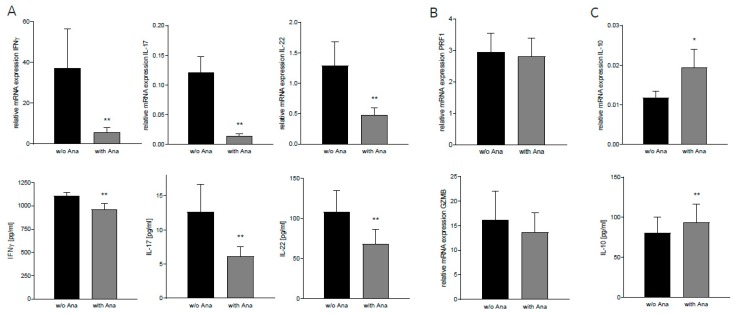
Anakinra reduced inflammatory signaling in lymphocytes co-cultured with T98G cells. T cells were isolated from PBMC after indirect co-culture with or without (w/o) anakinra (Ana). Protein levels in conditioned media from indirect co-cultures were assessed by enzyme-linked immunosorbent assay (ELISA). (**A**) mRNA and protein levels of proinflammatory cytokines IFNγ, IL-17, and IL-22 (mRNA: n = 13; protein: n = 10, *p* < 0.004). (**B**) mRNA expression of cytotoxic T-cell effector molecules PRF1 and GZMB (n = 9). (**C**) mRNA and protein level of anti-inflammatory cytokine IL-10 (mRNA: n = 13, *p* = 0.038; protein: n = 10, *p* = 0.002). * *p* < 0.05, ** *p* < 0.01.

Effective adaptive immunity is essential for long-term tumor control. On the other hand, inadequate proinflammatory cytokine secretion by T cells can support tumor growth and progression [7]. We assumed that anakinra might also affect these aspects of tumor-associated inflammation. We thus extracted T cells after indirect co-culture of PBMCs and GBM cells and analyzed expression levels of inflammatory cytokines that were (a) secreted by T cells and (b) involved in oncogenic signaling [25,26,27]. After administration of anakinra, we detected a marked decrease of proinflammatory *IFNγ*, *IL-17*, and *IL-22* mRNA expression (Figure 5A: *IFNγ*: −85.0% ± 45.4%, *IL-17*: −88.1% ± 21.7%, *IL-22*: −63.2% ± 22.0%, n = 13, *p* < 0.004) in these T cells as well as reduced protein levels in the respective conditioned medium (Figure 5A: IFNγ: −13.04% ±3.3, IL-17: −51.8% ± 20.8%, IL-22: −37.1% ± 8.5%, n = 10, *p* < 0.004). Notably, anakinra did not alter perforin (*PRF1*) or granzyme B (*GZMB*) levels in cytotoxic T cells (Figure 5B, n = 9), suggesting that CD8^+^-T-cell-mediated antitumor immunity remains unaffected after anakinra treatment. In addition, anakinra promoted gene expression and protein secretion of the anti-inflammatory cytokine IL-10 (Figure 5C: mRNA: +64.5% ± 30.0%, n = 13, *p* = 0.038; protein: +15.5% ± 5.1%, n = 10, *p* = 0.002).

## 3. Discussion

Tumor-promoting inflammation is an important driver of GBM progression. Inflammatory mediators are secreted by both tumor and immune cells in a mutually reinforcing manner [5,28]. In this study, we uncovered a new strategy to ameliorate this inflammatory feed-forward loop. We showed that the IL-1 antagonist anakinra puts the brake on inflammatory gene expression in both GBM and immune cells, and thereby attenuates GBM cell aggressiveness.

Interleukin-1β (IL-1β) is the “master regulator” of innate immunity and one of the most potent inflammatory cytokines. It is secreted by both immune and GBM cells [11,29], and treatment of GBM cells with IL-1β has been shown to enhance migration, invasion, and proliferation [17,30]. This more aggressive GBM phenotype has been attributed to increased levels of the tumor-promoting cytokine IL-6 and induction of STAT3 [31,32]. However, the actual role of IL-1β within the inflammatory networks of the tumor environment has not been investigated to date. We treated GBM cells and PBMCs with IL-1β and found dramatically increased expression levels of the proinflammatory and tumor-promoting mediators *COX2, IL-1β, CCL2,* and *IL-8* [33,34,35,36,37,38] in both GBM cells and PBMCs. Moreover, functional assays revealed a significant induction of GBM cell proliferation and migration. IL-1β is known to influence apoptosis of tumor cells via induction of STAT3 [11,20,21,22] Concordantly, we found a tendency towards up-regulation of *STAT3* and decreased apoptosis rates in GBM cells after IL-1β treatment (Figure S3). These results let us to assume that IL-1β might contribute to an inflammatory milieu that fosters GBM aggressiveness, and that blocking IL-1β-dependent pathways might be a promising strategy. Indeed, application of the IL-1 antagonist anakinra abrogated expression of the inflammatory gene signature and attenuated GBM cell proliferation and migration, and increased apoptosis rates.

The tumor microenvironment, however, is characterized by the presence of a plethora of inflammatory mediators. Thus, for more lifelike conditions, IL-1β stimulation was substituted by a newly established model of indirect PBMC/GBM cell co-culture in moderate hypoxia. (Moderate hypoxia is the prevalent condition within the vital, invasive zone of GBM tumors.) Surprisingly, sole incubation of GBM cells and PBMCs without any additional stimuli induced inflammatory gene expression profiles in both GBM cells and PBMCs, highly resembling those found in response to IL-1β stimulation. Concordantly, increased GBM proliferation rates and enhanced directed tumor cell migration towards a chemotactic stimulus were found. Apoptosis rates of GBM cells were not influenced in these indirect co-cultures. In fact, apoptosis was reduced after direct co-culturing of GBM cells with PBMCs. These findings underscore the role of direct cell–cell contact with T cells as an inductor of GBM escape mechanisms that prevent them from undergoing apoptosis [39,40].

In the setting of direct or indirect co-culture of immune cells with GBM, anakinra administration also mitigated proinflammatory gene expression and attenuated the inflammation-induced effects; GBM cell proliferation and migration were slowed down and apoptosis rate was increased.

GBM-associated inflammation is mounted by a dynamic “crosstalk” between tumor and immune cells [41,42,43]. In this regard, activated lymphocytes may be a double-edged sword. On one hand, their activity as gatekeepers of adaptive immunity is needed [41], but on the other hand, an inflammatory T-cell phenotype boosts tumor progression. In GBM, the proinflammatory cytokines IL-17 and IL-22 have been identified as cancer-promoting molecules activating oncogenic STAT3 signaling, thereby protecting GBM cells from apoptosis, promoting GBM proliferation and enhancing invasion [26,27,44]. IL-22 plays a particularly important role in immune cell–tumor interactions, as it is solely secreted by immune cells and exerts its effects only on non-hematopoietic cells [45]. Additionally, recent studies have revealed that IFNγ, formerly acknowledged as a prototypical anti-tumor mediator, might play a multi-functional role in cancer development by displaying tumor-enhancing properties [46,47]. We thus evaluated the influence of anakinra on T cells after co-cultivation with GBM cells, and found the expression and secretion of IFNγ, IL-17, and IL-22 to be strongly reduced. Moreover, anakinra treatment shifted T cells towards a more anti-inflammatory phenotype, hampering the secretion of proinflammatory and enhancing production of anti-inflammatory cytokines without affecting key molecules of anti-tumor immunity.

Taken together, our data underline the role of IL-1β as a hub of inflammatory signaling within the tumor microenvironment that drives the tumor-promoting crosstalk between GBM and adaptive immune cells, and thus promotes GBM aggressiveness. We have provided evidence that this detrimental feed-forward circuitry can be disrupted and a more benign GBM cell phenotype can be induced in vitro by administration of anakinra. However, the influence of anakinra on cells of innate immunity, e.g., macrophages/macroglia, in the context of GBM remains to be investigated in the future. 

The recombinant IL-1 receptor antagonist anakinra is currently approved for the treatment of rheumatoid arthritis (RA) and several rather rare autoinflammatory diseases, in which it is self-administered subcutaneously on a daily basis and is generally well tolerated, with skin reactions at the injection site as the most common treatment-associated side effect [48,49]. Particularly in RA, studies have demonstrated a decrease of disease-driving inflammatory cytokines IFNγ and IL-17 in patients treated with anakinra. These observed immunomodulating effects have been correlated with favorable clinical effects such as significant reduction of clinical symptoms and comorbidities like type 2 diabetes [50,51].

As anakinra is able to cross the blood–brain barrier and reaches therapeutically relevant concentration within the central nervous system [52,53,54], pharmacological interruption of tumor-associated inflammation by antagonizing IL-1 signaling might be an interesting new strategy by which to ameliorate GBM aggressiveness. Our study could provide the experimental basis for future clinical studies in this direction.

## 4. Materials and Methods 

### 4.1. Human Tissue Sample

GBM specimens were collected from patients undergoing open tumor resection (Supplementary Table S2). Brain Tumor Dissociation Kit and gentleMACS dissociator (Miltenyi, Bergisch-Gladbach, Germany) were used according to the manufacturer’s instructions. Cells were maintained in MACS Neuro Medium supplemented with 10% FCS, 2% L-Glutamine, 2% penicillin/streptomycin, and 2% Neuro Brew-21 without vitamin A (Miltenyi, Bergisch-Gladbach, Germany). The study was carried out following the rules of the Declaration of Helsinki of 1975, revised in 2013, and was approved by the local ethics committee of the Ludwig-Maximilians University Munich, approval no. 216/14. Written informed consent was obtained from all patients.

### 4.2. Cell Culture and Reagents

GBM cell lines U87 and T98G were purchased from the American Type Cell Culture Collection (ATCC, Manassas, VA, USA), and are routinely authenticated every year by PCR. Cells were cultured in Dulbecco’s modified Eagle’s medium (DMEM) (Gibco, NY, USA) with 10% fetal calf serum (FCS) (Biochrom GmbH, Berlin, Germany), 1% non-essential amino acids (Gibco, NY, USA), and 1% penicillin/streptomycin (Gibco, NY, USA). Cells were incubated at 37 °C and 5% CO_2_, in a humidified cell culture incubator (Binder, Tuttlingen, Germany), and passaged no more than 10 times. Interleukin-1β (IL-1β) was purchased from Miltenyi (Cat. No. 130-093-897, Bergisch-Gladbach, Germany) and used at the concentration of 10ng/mL. Anakinra (Kineret®, Swedish Orphan Biovitrum AB, Stockholm, Sweden) was used at a final concentration of 1 µg/mL.

### 4.3. PBMC Isolation

Peripheral blood mononuclear cells (PBMCs) were obtained from healthy donors by venipuncture. PBMC were isolated from Li-Heparin blood using Histopaque-1077 (Sigma-Aldrich, MO, USA) by density gradient centrifugation and automatically counted on a Vi-Cell (Beckman-Coulter, Brea, USA). Viability was >97% in all approaches. 

### 4.4. Co-Cultures

GBM cells were seeded in six well plates (Cellstar®, Greiner Bio-one, Austria) containing 2 mL DMEM. After adherence, medium was changed to 3 ml RPMI 1640 medium (Gibco), supplemented with 10% FCS, 1% L-Glutamine, 1% penicillin/streptomycin, and 1% HEPES buffer solution (Gibco), and PBMCs, stimulated with Human T-Activator CD3/CD28 Dynabeads (Thermo Fisher, Waltham, MA, USA) were added reaching a final GBM cell–PBMC ratio of 1:10. Cells were incubated in the present or absence of anakinra (1 µg/mL) under moderate hypoxic conditions in an airtight modular incubator chamber (Billups-Rothenberg, San Diego, USA) at 5% O_2_, 40 mmHg CO_2_ and 37 °C. For indirect co-culture experiments, GBM cells were separated from PBMCs by cell culture inserts (pore diameter: 0.4 μm) (Greiner Bio-one, Austria). After 24 h or 48 h cells, were harvested for further analyses. Cell culture supernatant was aliquoted and stored at −80 °C. Supernatant from direct co-cultures were subsequently subjected to native GBM cells, seeded the previous day. After addition of anakinra, cells were also incubated in moderate hypoxia for 48 h. 

### 4.5. T-Cell Isolation

PBMC from indirect co-culture approaches were harvested after 48 h for T-cell isolation. Purified T cells were obtained by microbead separation using the Pan T Cell Isolation Kit (Cat. No. 130-096-535, Miltenyi, Bergisch-Gladbach, Germany) and the AutoMACS Pro Separator (Miltenyi, Bergisch-Gladbach, Germany) according to the manufacturer’s instructions.

### 4.6. RNA Extraction and cDNA Synthesis 

Total RNA was extracted from cell lysates using the RNAqueous Isolation Kit (Ambion, Waltham, MA, USA) and the TURBO DNA-free Kit (Invitrogen, Waltham, MA, USA) to remove DNA contamination. RNA sample measurements were conducted using a NanoDrop 2000 spectrophotometer (Thermo Scientific, Waltham, MA, USA). cDNA was synthesized using Oligo-dT Primers, Random Hexamers (Qiagen, Venlo, Netherlands), dNTPs, RNAse OUT, and Superscript® III Reverse Transcriptase (Invitrogen, Waltham, MA, USA) according to the manufacturer’s instructions.

### 4.7. Quantitative RT- PCR

Quantitative polymerase chain reaction (qPCR) was performed using a LightCycler480 (Roche Diagnostics, Penzberg, Germany) as previously described [55]. TATA Box Binding Protein (TBP) and Succinate Dehydrogenase Subunit A (SDHA) were used as reference genes. All assays were designed to be intron spanning. Primers (Metabion, Martinsried, Germany) and UPL Probe numbers (Roche Diagnostics, Penzberg, Germany) are provided in Supplementary Table S1.

### 4.8. Flow Cytometry

All cytometric analyses were performed using a BD FACSCanto II flow cytometer (BD, NJ, USA). Data were analyzed by FlowJo software, version v10 (FlowJo, Ashland, USA). To assess cell proliferation rate, cells were harvested and stained with Alexa Fluor 488 anti-human Ki-67 antibody (Cat. No. 350507, Clone: Ki-67, BioLegend, CA, USA). Alexa Fluor 488 Mouse IgG1, κ antibody (Cat. No. 400134, Clone: MOPC-21, BioLegend, CA, USA) was used as isotype control. Staining process for Ki-67 was conducted following the manufacturer’s protocol. For intracellular staining, cells were permeabilized and fixated in 70% ethanol (−20 °C) for 60 minutes, washed with BioLegend Cell Staining Buffer, and subsequently incubated with the respective antibody for 30 minutes. Apoptosis assay was conducted using Violet Chromatin Condensation/Dead Cell Apoptosis Kit (Cat. No. A35135, Invitrogen, Waltham, MA, USA). A total of 30,000 events were recorded and analyzed respectively.

### 4.9. Chemotaxis Assay

The chemotaxis assay was conducted using ibidi μ-Slides Chemotaxis (ibidi, Martinsried, Germany) following the manufacturer’s application guide. Either T98G/primary GBM cells, stimulated with IL-1β under hypoxic conditions or T98G from indirect co-cultures, incubated in the absence or presence of anakinra (1 µg/mL), were loaded into slides. After cell adhesion, media in the observation channel was changed to the respective media without FCS and FCS was applied to one of the adjacent reservoirs, serving as a chemoattractant. Cell movement in the central channel was observed under constant incubation at 37 °C using an inverted microscope (Zeiss, Jena, Germany; magnification: 10×). Pictures were taken automatically every 10 minutes for 24 hours. Cell tracking was performed manually using ImageJ Manual Tracking Plugin (ImageJ, NIH, USA). Data were analyzed using the Chemotaxis and Migration Tool (ibidi, Martinsried, Germany).

### 4.10. Enzyme-Linked Immunosorbent Assay

Supernatants harvested from GBM–PBMC indirect co-cultures were immediately stored at −80 °C. Frozen supernatants were thawed slowly on ice. Measures of 100 µl of supernatant were subjected to 96 well enzyme-linked immunosorbent assays (ELISA). Cytokine levels were measured using the respective ELISA MAX™ Deluxe Set according to the manufacturer’s protocols (Interferon γ (IFNγ) ELISA Kit, Cat. No. 430107, IL-22 ELISA Kit, Cat. No. 434504, IL-17 ELISA Kit, Cat. No.435707, IL-10 ELISA Kit, Cat. No. 430604, BioLegend, San Diego, CA, USA). Optical density was determined using a FilterMax F3 MultiMode Microplate Reader (Molecular Devices, Munich, Germany) at a wavelength of 450 nm.

### 4.11. Sodium Dodecyl Sulfate Polyacrylamide Gel Electrophoresis 

Cells were lysed in protein lysis buffer containing protease and phosphatase inhibitors (Cell Signaling, Danvers, MA, USA). Protein concentrations were measured using a Pierce^TM^ BCA Protein Assay Kit (Thermo Fisher Scientific, Waltham, MA, USA), following the manufacturer’s instructions. A total 15 µg of protein extract was loaded into each lane of 12% sodium dodecyl sulfate polyacrylamide gel electrophoresis gels, electrophoresed, and subsequently transferred onto PVDF membranes. Nonspecific binding was blocked using TBS-Tween-20 (TBST) solution containing 5% non-fat milk. Antibodies for BCL-2 (Clone: D55G8, Cat. No. 4223) and β-Actin (Clone: 13E5, Cat. No. 4970) were diluted in TBST with 1% non-fat milk. β-Actin was used as loading control. Immunoreactivity was assessed using horseradish-peroxidase-labeled goat anti-rabbit antibodies (Cell Signaling, Danvers, MA, USA). Blots were visualized by digital imaging using Clarity^TM^ Western ECL Substrate kit (Bio-Rad, CA, USA). Densitometric analysis was performed using ImageJ (ImageJ, NIH, USA).

### 4.12. Transwell Invasion Assay

Transwell invasion assays were performed using the CytoSelect^TM^ 24-Well Cell Invasion Assay (Cell Biolabs, Inc. San Diego, CA, USA), following the manufacturer’s instructions. T98G cells, stimulated with IL-1β, in the absence or presence of anakinra (1 µg/mL), were seeded into rehydrated cell culture inserts (pore diameter: 8 μm) in serum-free DMEM. DMEM containing 10% FCS was added to the lower well of the invasion plate. After incubation for 24 h at 37 °C, non-invasive cells were removed from the inside of the inserts with cotton swabs. Invasive cells were stained, lysed, and the optical densities were determined using a FilterMax F3 MultiMode Microplate Reader (Molecular Devices, Munich, Germany) at a wavelength of 560 nm.

### 4.13. Wound Healing Migration Assay

T98G cells stimulated with IL-1β, in the absence or presence of anakinra (1 µg/mL) were seeded in 24 well plates at a density of 200,000 cells/well (Cellstar®, Greiner Bio-one, Austria) and incubated at 37 °C with 5% CO_2_ in FCS-free media. After 24 h, a pipette tip was used to manually scratch a defined wound through the monolayer of cells. Cell debris was removed by washing the cells once with 1 mL of media. Washing media was replaced with DMEM containing 10% FCS and cells were incubated for 24 h. Pictures were obtained using an inverted microscope (Carl Zeiss, Jena, Germany).

### 4.14. Statistics

All data are presented as mean ± SEM unless stated otherwise. Statistical analysis was conducted using GraphPad Prism 7 software. All datasets were tested for Gaussian distribution, and *p*-values were calculated using Student’s *t*-test or Wilcoxon signed rank test. *p* < 0.05 was considered statistically significant (* *p* < 0.05, ** *p* < 0.01, *** *p* < 0.001, n.s. = not significant). All experiments were conducted at least three times.

## 5. Conclusions

In this study, we underscored the role of IL-1β within the GBM signaling network. In human glioma, IL-1β-related inflammation promotes cancer malignancy and is markedly amplified through the crosstalk of cancer cells with surrounding immune cells.

By blocking this self-perpetuating circuit, our data highlighted the role of IL-1 receptor antagonist anakinra as a new potential therapeutic agent in GBM treatment, which merits further investigation.

## Supplementary Materials

The following are available online at https://www.mdpi.com/2072-6694/12/2/433/s1, Figure S1: (A) Dose-finding analysis of IL-1β. (B) Dose-finding analysis of anakinra. (C) mRNA expression of proinflammatory IL-1β, COX2, IL-8 and IL-6 in U87 cells, Figure S2: Analysis of chemotaxis time-lapse microscopy by single-cell tracking of primary GBM cells, Figure S3: (A) mRNA expression of STAT3 in T98G cells after stimulation with IL-1β in the present or absence of anakinra, as analyzed by qRT-PCR. (B) Percentage of apoptotic T98G cells after stimulation with IL-1β, in the present or absence of anakinra, as measured by flow cytometry, Table S1: Primer sequences for qRT-PCR, Table S2: Patient characteristics.
