# Supplementary material for: The IL-1 Antagonist Anakinra Attenuates Glioblastoma Aggressiveness by Dampening Tumor-Associated Inflammation
Source: Cancers (Basel). 2020 Feb 13;12(2):433. doi: 10.3390/cancers12020433 (PMC7072290; doi:10.3390/cancers12020433)
Supplement: Supplementary file 1 [file cancers-12-00433-s001.zip › Suppl_Fig_1.pdf]

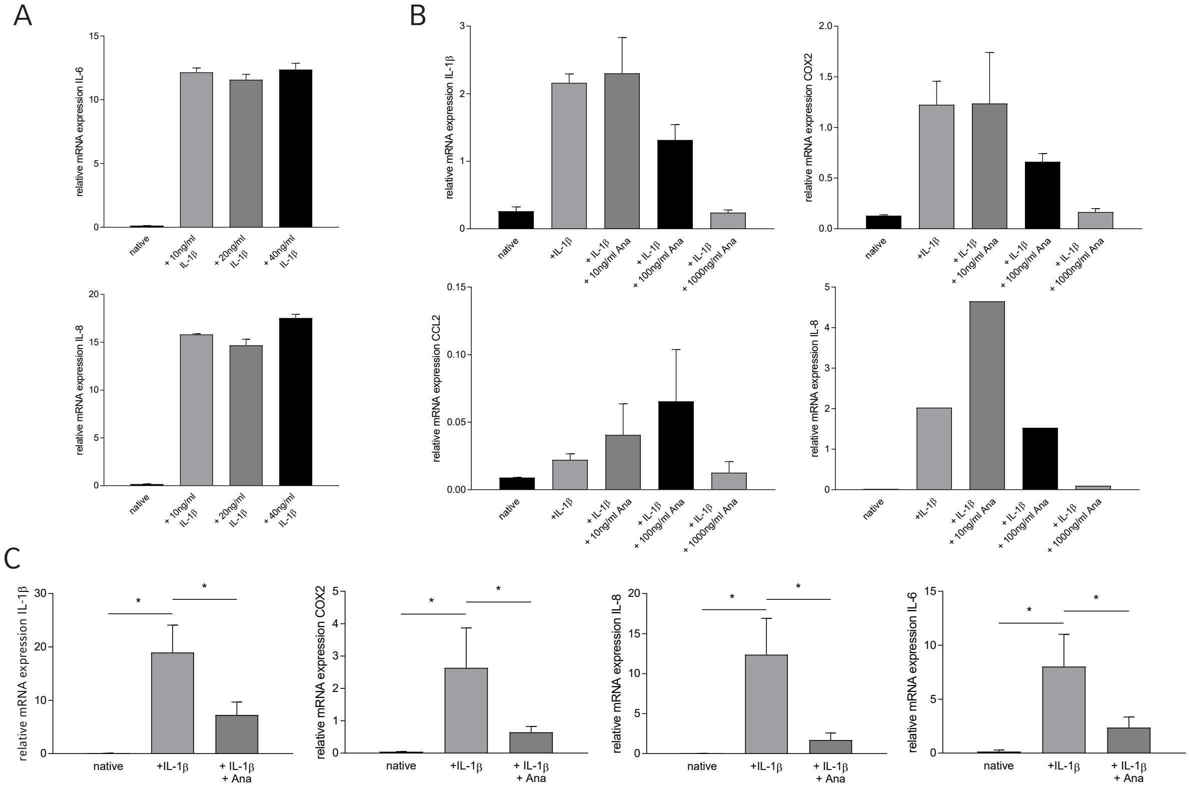

Figure S1. (A) Dose-finding analysis of IL-1β. mRNA expression of *IL-6* and *IL-8* in T98G cells after stimulation with IL-1β (10, 20 or 40ng/ml) for 48h, as analyzed by qRT-PCR (n=4). **(B)** Dose-finding analysis of anakinra. mRNA expression of *IL-1β, COX2, CCL2* and *IL-8* in T98G cells after stimulation with IL-1β (10ng/ml) and different doses of anakinra for 48h, as analyzed by qRT-PCR (n=4). **(C)** mRNA expression of proinflammatory *IL-1β, COX2, IL-8* and *IL-6* in U87 cells, as quantified by qRT-PCR. U87 cells were stimulated with IL-1β (10ng/ml) in the presence or absence of anakinra (1μg/ml), for 48h (n=6). *$p < 0.05$.
