# Supplementary material for: The IL-1 Antagonist Anakinra Attenuates Glioblastoma Aggressiveness by Dampening Tumor-Associated Inflammation
Source: Cancers (Basel). 2020 Feb 13;12(2):433. doi: 10.3390/cancers12020433 (PMC7072290; doi:10.3390/cancers12020433)
Supplement: Supplementary file 1 [file cancers-12-00433-s001.zip › Suppl_Fig_2.pdf]

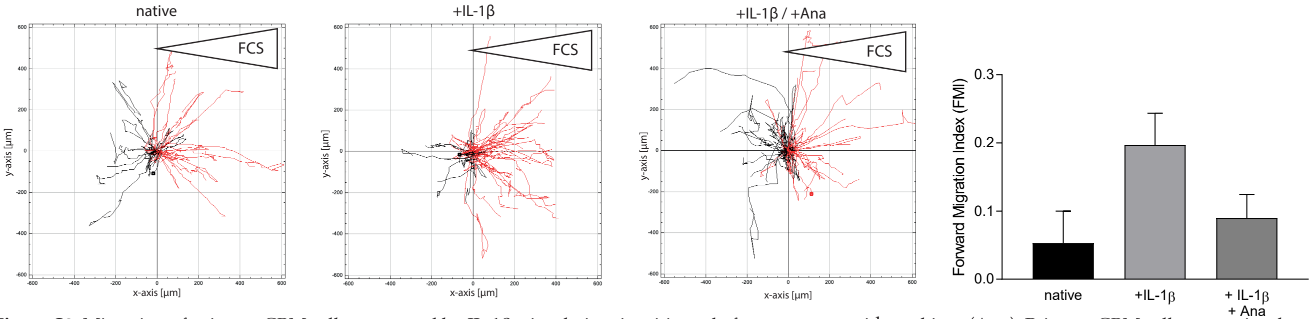

Figure S2. Migration of primary GBM cells, promoted by IL-1β stimulation, is mitigated after treatment with anakinra (Ana). Primary GBM cells were stimulated with IL-1β in the presence or absence of anakinra. Analysis of chemotaxis time-lapse microscopy by single-cell tracking. Primary GBM cells were incubated with FCS as a chemotactic stimulus (right reservoir). At least 40 cells were tracked. One representative example of three independent experiments is shown (left panel, n=3). Analysis of Forward Migration Index (FMI, coordinate of a cell in the indicated direction (x-axis) divided by the accumulated distance of its paths, representing efficiency of forward migration) (right panel, n=3)
