# Supplementary material for: The IL-1 Antagonist Anakinra Attenuates Glioblastoma Aggressiveness by Dampening Tumor-Associated Inflammation
Source: Cancers (Basel). 2020 Feb 13;12(2):433. doi: 10.3390/cancers12020433 (PMC7072290; doi:10.3390/cancers12020433)
Supplement: Supplementary file 1 [file cancers-12-00433-s001.zip › Suppl_Fig_3.pdf]

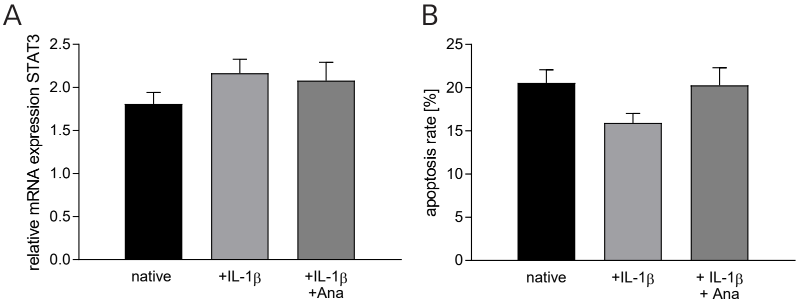

Figure S3. (A) mRNA expression of *STAT3* in T98G cells after stimulation with IL-1β in the present or absence of anakinra, as analyzed by qRT-PCR (n=4, p=n.s.). **(B)** Percentage of apoptotic T98G cells after stimulation with IL-1β, in the present or absence of anakinra, as measured by flow cytometry (n=4, p=n.s.).
