# Supplementary material for: The IL-1 Antagonist Anakinra Attenuates Glioblastoma Aggressiveness by Dampening Tumor-Associated Inflammation
Source: Cancers (Basel). 2020 Feb 13;12(2):433. doi: 10.3390/cancers12020433 (PMC7072290; doi:10.3390/cancers12020433)
Supplement: Supplementary file 1 [file cancers-12-00433-s001.zip › Suppl. Table 1.pdf]

Table S1. qRT-PCR primer sequences and assay characteristics

Target	UPL probe number	Primer sequence
TBP	87	forward: 5′-GAACATCATGGATCAGAACAACA-3′
		reverse: 5′-ATAGGGATTCCGGGAGTCAT-3′
SDHA	132	forward: 5′-GAGGCAGGGTTTAATACAGCA-3′
		reverse: 5′-CCAGTTGTCCTCCTCCATGT-3′
IL-1β	41	forward: 5′-GAGGCACAAGGCACAACAG-3′
		reverse: 5′-CCATGGCTGCTTCAGACAC-3′
CCL2	40	forward: 5′-AGTCTCTGCCGCCCTTCT-3′
		reverse: 5′-GTGACTGGGGCATTGATTG-3′
COX-2	2	forward: 5′-GCTTTATGCTGAAGCCCTATGA-3′
		reverse: 5′-TCCAACTCTGCAGACATTTCC-3′
BCL-2	6	forward: 5′-ACAGAGGATCATGCTGTACTTAAAAA-3′
		reverse: 5′-TTATTTCATGAGGCACGTTATTATTAG-3′
STAT3	4	forward: 5′-GGCCATCTTGAGCACTAAGC-3′
		reverse: 5′-CGGACTGGATCTGGGTCTTA-3′
IL-22	6	forward: 5′-CAACAGGCTAAGCACATGTCA-3′
		reverse: 5′-ACTGTGTCCTTCAGCTTTTGC-3′
IL-17	8	forward: 5′-TGGGAAGACCTCATTGGTGT-3′
		reverse: 5′-GGATTTCGTGGGATTGTGAT-3′
IFNγ	21	forward: 5′-GGCATTTTGAAGAATTGGAAAG-3′
		reverse: 5′-TTTGGATGCTCTGGTCATCTT-3′
IL-10	67	forward: 5′-TGCCTTCAGCAGAGTGAAGA-3′
		reverse: 5′-GCAACCCAGGTAACCCTTAAA-3′
GZMB	37	forward: 5′-GGGGGACCCAGAGATTAAAA-3′
		reverse: 5′-CCATTGTTTCGTCCATAGGAG-3′
PRF1	26	forward: 5′-CACTCACAGGCAGCCAACT-3′
		reverse: 5′-GGGAGTGTGTACCACATGGA-3′
BAX	69	forward: 5′-CATCATGGGCTGGACATTG-3′
		reverse: 5′-GGGACATCAGTCGCTTCAGT-3′
IL-8		Roche RealTime Ready Single Assay ID 103136
