# Supplementary material for: The IL-1 Antagonist Anakinra Attenuates Glioblastoma Aggressiveness by Dampening Tumor-Associated Inflammation
Source: Cancers (Basel). 2020 Feb 13;12(2):433. doi: 10.3390/cancers12020433 (PMC7072290; doi:10.3390/cancers12020433)
Supplement: Supplementary file 1 [file cancers-12-00433-s001.zip › Suppl. Table 2.pdf]

Table S2. Patient characteristics

patient	MGMT-status	IDH-status	age [years]	gender
1	unmethylated	wild-type	66	male
2	methylated	wild-type	65	male
3	partially methylated	wild-type	77	female
4	methylated	wild-type	51	female
5	unmethylated	wild-type	57	female
6	unmethylated	wild-type	55	male
